# Experiences of maternal distress and early childhood development amid climate-related stress in Benue state, Nigeria: a qualitative study

**DOI:** 10.3389/fpubh.2026.1940042

**Published:** 2026-10-28

**Authors:** Ngozi Idemili-Aronu, John Jemisenia, Deborah Aluh, Echezona Edozie Ezeanolue, Rathi Ravindrarajah, Amy Blakemore, Zahra Mulla, Tung Le, Asri Maharani, Jahanara Miah

**Affiliations:** 1 IVAN Research Institute, Enugu, Nigeria; 2 Department of Sociology/Anthropology, University of Nigeria, Nsukka, Nigeria; 3 Department of Psychiatry, University of Oxford, Oxford, United Kingdom; 4 College of Medicine, University of Nigeria, Nsukka, Nigeria; 5 Division of Nursing, Midwifery and Social Work, University of Manchester, Manchester, United Kingdom; 6 Division of Medical Education, University of Manchester, Manchester, United Kingdom; 7 Department of General Planning, Hanoi Medical University Hospital, Hanoi Medical University, Hanoi, Vietnam; 8 Faculty of Biology, Medicine and Health, University of Manchester, Manchester, United Kingdom

**Keywords:** climate change, early childhood development, flooding, heat, maternal mental health, Nigeria

## Abstract

**Background:**

Climate-related flooding and extreme heat affect maternal and child wellbeing in low-and middle-income countries. Although evidence suggests that climate-related events can affect mental health and child wellbeing, little is known about how these experiences are described in flood-prone settings. This study explores participants’ accounts of flooding, extreme heat, and household vulnerability in Benue State and their perceived implications for maternal mental health and early childhood development.

**Methods:**

A qualitative descriptive design was used. Data were collected through six focus group discussions and six semi-structured interviews with 54 mothers, health workers, community leaders, and policymakers and analysed using reflexive thematic analysis.

**Results:**

Participants described flooding as a recurrent threat shaped by spatial vulnerability, poor drainage, and inadequate land-use planning. Participants described flooding in relation to disrupted routines, livelihoods, food and water security, and caregiving; mothers and health workers also described extreme heat as disrupting sleep and caregiving. Participants described maternal psychological distress associated with uncertainty, household disruption, economic losses, and caregiving demands. Participants described challenges affecting children’s nutrition, healthcare access, immunisation, learning, safety, and social development. Community coping strategies included collective action and social support, while participants described gaps in formal responses.

**Conclusion:**

The findings highlight interconnected experiences of climate-related disruption, maternal distress, caregiving, and conditions relevant to early childhood wellbeing. Responses could support continuity of health services, nutrition, early learning, safe environments, psychosocial support, and community participation.

## Introduction

Nigeria is highly vulnerable to climate change, with flooding creating serious health risks for communities in Nigeria. Around 24.7 million people (about 8.3% of the population) were exposed to flooding in 2015, with exposure increasing over time (1). Floods damage water and sanitation systems, which can lead to waterborne diseases, malnutrition, and people being forced to leave their homes (2, 3). In addition, flooding has been linked to increased incidence of diseases, such as malaria, and diarrhoeal illnesses, malnutrition, and heightened psychological stress (2). Beyond flooding, other climate-related hazards further compound health risks; for example, heatwaves can lead to exhaustion and cardiovascular complications, particularly for vulnerable groups (4, 5), while droughts reduce agricultural productivity, worsening food shortages and food insecurity for the local communities (6). Together, flooding and extreme heat represent climate-related stressors that can affect health and living conditions across affected communities.

Beyond the physical health consequences of climate-related hazards, implications for maternal mental health necessitate closer attention, particularly in low-and middle-income countries (LMICs). Maternal mental health is a particular concern as the literature suggests perinatal mental health problems are more prevalent in LMIC settings, with an estimated 15.6% of women experiencing mental health problems during pregnancy, and 19.8% after childbirth (7). A more recent systematic review and meta-analysis of 589 studies across 51 LMICs estimated the pooled prevalence of perinatal depression at 24.7% (8), and emerging evidence suggests that climate-related events, including floods and storms, can further exacerbate these vulnerabilities (9, 10). Extreme heat has likewise been associated with disturbed sleep, irritability, and poorer mental health, though evidence from low-resource, high-exposure settings remains scarce (10).

Maternal mental health is also closely linked to early childhood development (ECD), which refers to children’s cognitive, physical, language, and socio-emotional development during the early years of life (11). Poor maternal mental health, particularly depression, has been associated with weaker mother–child attachment, suboptimal child development outcomes and poorer nutritional status (12, 13). Despite strong evidence supporting these relationships, there remains a significant gap in understanding how climate-related events and their associated stressors are experienced in relation to maternal mental health and early childhood development in the Nigerian context.

While quantitative research examining the epidemiological associations between climate change, mental health, and child development provides important evidence of the scale of these challenges (14), it is limited in its ability to capture lived experiences, contextual meanings, and the structural conditions that shape individual and community responses to flooding. Qualitative approaches are therefore valuable for exploring how climate-related exposures are experienced and described in relation to maternal mental health and child wellbeing, and for exploring the complex interplay of social, community, and individual factors that underpin wellbeing (15, 16). However, qualitative evidence in this area remains limited, constraining a more comprehensive understanding of these relationships (14).

This paper reports on the qualitative component of a broader mixed-methods study examining the links between climate-related flooding, maternal mental health, and early childhood development in Nigeria. The qualitative study aimed to explore community experiences of flooding, extreme heat, and household vulnerability in relation to maternal mental health and ECD, and to generate contextually grounded insights to inform intervention adaptation strategies and future research priorities.

## Materials and methods

### Study design

This study used a qualitative descriptive design to explore people’s experiences of flooding, extreme heat and other climate stressors, maternal mental health, and early childhood development in Benue State, Nigeria, with combined semi-structured interviews and focus group discussions. This design was used to compare individual experiences with shared group perspectives, allowing for stronger analysis through triangulation. This approach allows for the collection of rich, detailed data that reflects the complexity of everyday life to understand real-life experiences directly from participants and stay close to how people describe their own situations (17, 18).

### Study setting

This study was conducted in Benue State, Nigeria, a region that is highly vulnerable to seasonal flooding, particularly along the Benue and Katsina-Ala river basins. Benue State experiences frequent and severe flooding events affecting communities across the river basins, leading to major consequences such as population displacement, loss of agricultural livelihoods, and widespread damage to infrastructure (19, 20). Food insecurity is a further concern, as flooding reduces food production and worsens household food insecurity (6). While flooding is the most prominent local climate hazard, Nigeria also experiences important heat-related health risks (21, 22). Benue State provides a relevant and important setting for this study due to its high exposure to flooding and associated social and health challenges.

### Participants and sampling

Participants were selected using purposive sampling to ensure a wide range of perspectives on flooding, maternal mental health, and early childhood development. The study included four key groups: mothers of children aged 0–5 years who had direct experience of flooding; frontline health workers involved in delivering maternal and child health services; religious and community leaders who influence community beliefs and practices related to health; and local government officials (who we referred to as policy makers) responsible for disaster preparedness, social welfare, and health governance. This multi-stakeholder approach was used to capture different levels of experience and insight, from individual and household impacts to healthcare delivery, community influence, and policy-level responses. The use of multiple stakeholder groups was intended to capture perspectives from households, health services, community structures, and local governance.

Participants were recruited from communities across three Local Government Areas (LGAs) in Benue State: Otukpo, Gboko, and Makurdi (the state capital). Potential participants were invited through recruitment flyers circulated in person via community and professional networks in Benue State. Those who expressed interest were provided with a participant information sheet and screened for eligibility. Those who met the inclusion criteria and wished to participate were invited to provide informed consent before taking part. Participants were selected across different backgrounds, including variation in location, flood exposure, socioeconomic status, and professional roles, to capture diverse perspectives. A total of 54 participants were included in the study across three Local Government Areas (LGAs), namely Makurdi, Gboko, and Otukpo. The sample comprised 24 mothers and 24 health workers who participated in focus group discussions (FGDs), with one FGD conducted for each stakeholder group in each LGA and eight participants per FGD. In addition, six individual interviews were conducted across the three LGAs: three policymakers participated in key informant interviews (KIIs), with one policymaker interviewed in each LGA, while three community leaders participated in individual in-depth interviews (IDIs), with one community leader interviewed in each LGA. Thus, the study comprised six FGDs involving 48 participants (24 mothers and 24 health workers) and six individual interviews involving six participants (three policymakers and three community leaders), providing perspectives from four stakeholder groups across the three study locations.

The sample size and composition were predetermined *a priori* based on the study design, which was structured to obtain perspectives from four stakeholder groups across three LGAs. Data saturation was not used as a criterion, as it is not consistent with reflexive thematic analysis (23); instead, adequacy was considered in terms of information power (24), given the focused aim, specific sample and quality of dialogue. For each LGA, one focus group discussion comprising eight mothers and one focus group discussion comprising eight health workers were conducted, while one policymaker was purposively selected for a key informant interview and one community leader for an individual in-depth interview. The choice of eight participants per focus group was based on established methodological guidance for focus group composition (25). This structure resulted in 18 participants per LGA and a total sample of 54 participants across the three study locations. During each interview or focus group discussion, interviewers introduced themselves, reminded participants of the study aims and objectives and their right to withdraw consent, and invited questions before proceeding.

Overall, 54 participants were included in the study (Tables 1–3). Among mothers (Table 1), 62.5% were aged 30–39 years, 75% had secondary education and half of the mothers reported trading as their main source of income. Nearly all had experienced flooding in the preceding 2 years (95.8%), with 70.8% reporting flood-related displacement. Health workers (Table 2) were predominantly female (79.2%), mainly Community Health Extension Workers (50.0%), and largely employed in Primary Health Centres (58.3%). Among policymakers and community leaders (Table 3), all were male, all engaged in maternal and child health programmes, and half reported direct involvement in flood response and policy activities (50.0%).

**Table 1:** Demographic characteristics of mothers participating in the FGDs across Makurdi, Gboko, and Otukpo LGAs.

Characteristic	Makurdi (*n* = 8)	Gboko (*n* = 8)	Otukpo (*n* = 8)
Age band (years)			
20–29	2 (25.0)	2 (25.0)	1 (12.5)
30–39	5 (62.5)	5 (62.5)	5 (62.5)
≥40	1 (12.5)	1 (12.5)	2 (25.0)
Marital status			
Married	8 (100.0)	8 (100.0)	8 (100.0)
Number of children			
1 child	0 (0.0)	3 (37.5)	3 (37.5)
2–3 children	4 (50.0)	5 (62.5)	3 (37.5)
≥4 children	4 (50.0)	0 (0.0)	2 (25.0)
Age of youngest child			
<1 year	0 (0.0)	1 (12.5)	6 (75.0)
1–2 years	3 (37.5)	4 (50.0)	1 (12.5)
3–5 years	5 (62.5)	3 (37.5)	1 (12.5)
Education level			
No formal education	0 (0.0)	0 (0.0)	1 (12.5)
Primary	0 (0.0)	1 (12.5)	1 (12.5)
Secondary	7 (87.5)	5 (62.5)	6 (75.0)
Tertiary	1 (12.5)	2 (25.0)	0 (0.0)
Main household income source			
Farming	2 (25.0)	5 (62.5)	1 (12.5)
Trading	4 (50.0)	3 (37.5)	5 (62.5)
Paid employment	2 (25.0)	0 (0.0)	0 (0.0)
Other	0 (0.0)	0 (0.0)	2 (25.0)
Residence type			
Urban	8 (100.0)	1 (12.5)	1 (12.5)
Semi-urban	0 (0.0)	7 (87.5)	4 (50.0)
Rural	0 (0.0)	0 (0.0)	3 (37.5)
Experienced flooding in past 2 years			
Yes	8 (100.0)	7 (87.5)	8 (100.0)
No	0 (0.0)	1 (12.5)	0 (0.0)
Displacement due to flooding			
Yes	8 (100.0)	6 (75.0)	3 (37.5)
No	0 (0.0)	2 (25.0)	5 (62.5)

Demographic characteristics of mothers who participated in the focus group discussions, including age, educational attainment, occupation, flooding experience, displacement status, and other relevant socioeconomic characteristics.

**Table 2:** Demographic and service characteristics of health workers participating in the FGDs across Makurdi, Gboko, and Otukpo LGAs.

Characteristic	Makurdi (*n* = 8)	Gboko (*n* = 8)	Otukpo (*n* = 8)
Age category (years)			
20–29	1 (12.5)	2 (25.0)	0 (0.0)
30–39	2 (25.0)	3 (37.5)	3 (37.5)
40–49	2 (25.0)	2 (25.0)	3 (37.5)
50–59	2 (25.0)	1 (12.5)	2 (25.0)
≥60	1 (12.5)	0 (0.0)	0 (0.0)
Gender			
Female	6 (75.0)	5 (62.5)	8 (100.0)
Male	2 (25.0)	3 (37.5)	0 (0.0)
Marital status			
Married	4 (50.0)	1 (12.5)	6 (75.0)
Single	2 (25.0)	4 (50.0)	1 (12.5)
Widowed	0 (0.0)	2 (25.0)	1 (12.5)
Divorced/separated	2 (25.0)	1 (12.5)	0 (0.0)
Professional cadre			
CHEW/community health worker	5 (62.5)	5 (62.5)	2 (25.0)
Nurse/midwife	2 (25.0)	0 (0.0)	2 (25.0)
Laboratory staff	1 (12.5)	1 (12.5)	0 (0.0)
Environmental health officer	0 (0.0)	0 (0.0)	1 (12.5)
Social worker	0 (0.0)	0 (0.0)	1 (12.5)
Public health officer	0 (0.0)	1 (12.5)	0 (0.0)
Other	0 (0.0)	1 (12.5)	2 (25.0)
Highest qualification			
Certificate	3 (37.5)	1 (12.5)	0 (0.0)
Diploma	2 (25.0)	3 (37.5)	4 (50.0)
Bachelor’s degree	0 (0.0)	3 (37.5)	1 (12.5)
Master’s degree	2 (25.0)	0 (0.0)	1 (12.5)
Doctorate	1 (12.5)	0 (0.0)	0 (0.0)
Other/unspecified	0 (0.0)	1 (12.5)	2 (25.0)
Years of professional experience			
<5 years	1 (12.5)	0 (0.0)	0 (0.0)
5–10 years	2 (25.0)	4 (50.0)	2 (25.0)
11–15 years	1 (12.5)	1 (12.5)	4 (50.0)
16–20 years	1 (12.5)	1 (12.5)	1 (12.5)
>20 years	3 (37.5)	2 (25.0)	1 (12.5)
Type of facility			
Primary health centre	8 (100.0)	4 (50.0)	2 (25.0)
General hospital	0 (0.0)	4 (50.0)	2 (25.0)
NGO/community-based facility	0 (0.0)	0 (0.0)	2 (25.0)
Teaching hospital	0 (0.0)	0 (0.0)	1 (12.5)
Mixed facility affiliation	0 (0.0)	0 (0.0)	1 (12.5)
Location of practice			
Urban	8 (100.0)	5 (62.5)	1 (12.5)
Semi-urban	0 (0.0)	2 (25.0)	7 (87.5)
Rural	0 (0.0)	1 (12.5)	0 (0.0)
Main population served			
Pregnant women	5 (62.5)	1 (12.5)	1 (12.5)
Mothers and children	0 (0.0)	0 (0.0)	1 (12.5)
Children (0–5 years)	0 (0.0)	0 (0.0)	1 (12.5)
General population	3 (37.5)	3 (37.5)	4 (50.0)
Mixed populations	0 (0.0)	4 (50.0)	1 (12.5)
Role in MCH services			
Direct service delivery	7 (87.5)	4 (50.0)	3 (37.5)
Supervisory and direct service	1 (12.5)	0 (0.0)	3 (37.5)
Outreach/community services	0 (0.0)	4 (50.0)	1 (12.5)
Administrative role	0 (0.0)	0 (0.0)	0 (0.0)
Mixed/other roles	0 (0.0)	0 (0.0)	1 (12.5)

Demographic and professional characteristics of health workers who participated in the focus group discussions, including age, gender, professional cadre, years of experience, health facility level, and area of practice.

**Table 3:** Demographic characteristics of policymakers/key informants and community leaders participating in the study (*N* = 6).

Characteristic	*n*	%
Participant category		
Policymakers/key informants	3	50.0
Community leaders	3	50.0
Study location		
Makurdi	2	33.3
Gboko	2	33.3
Otukpo	2	33.3
Gender		
Male	6	100.0
Female	0	0.0
Age group (years)		
30–39	1	16.7
40–49	1	16.7
50–59	3	50.0
≥60	1	16.7
Institutional affiliation		
Government health sector	3	50.0
Community/traditional institutions	3	50.0
Government level among policymakers (*n* = 3)		
State government	2	66.7
Local government	1	33.3
Years in current role		
<5 years	1	16.7
5–10 years	4	66.7
>10 years	1	16.7
Involvement in flood/disaster response or policy		
Direct involvement	3	50.0
Occasional involvement	2	33.3
Limited/no direct involvement	1	16.7
Maternal and child health programme involvement		
Yes	6	100.0
No	0	0.0
Mental health policy involvement		
Yes	2	33.3
No	4	66.7

### Data collection

Data collection took place from 23rd February 2026 to 20th March 2026 and was led by NIA, a female medical sociologist with extensive qualitative research experience, supported by JJ, a male medical sociologist with experience in qualitative data collection. Data collection was undertaken in local languages (Tiv, Idoma, Nigerian Pidgin) and English. Research assistants undertaking data collection had prior experience of working in local communities and attended two bespoke orientation training sessions, which focused on the study objectives, ethical considerations, interviewing techniques, use of data collection tools, and facilitation of focus group discussions and interviews.

Focus group discussions with mothers were conducted in private community meeting spaces, while focus group discussions with health workers and individual interviews with policymakers and community leaders were conducted in health facilities, private offices, or other appropriate private settings. Sessions lasted 40–60 min; one interviewer facilitated each discussion or interview while the second took detailed notes in addition to the audio recording and documented contextual observations. Researcher reflections were completed immediately afterwards.

For both focus group discussions and one-to-one interviews, data collection was conducted using semi-structured topic guides, which were translated into relevant local languages (Tiv, Idoma, Nigerian Pidgin, and English), piloted prior to use, and iteratively refined throughout the study to improve clarity and contextual relevance. The mothers’ and health workers’ topic guides explicitly explored both flooding and extreme heat and their implications for maternal mental health, caregiving, and early childhood development, whereas the community leader and policymaker guides focused primarily on flooding, infrastructure, and institutional response; this design is reflected in the relative prominence of heat across participant groups in the findings.

All one-to-one interviews and focus group discussions were audio-recorded with participant consent, transcribed verbatim, and anonymised during transcription. Quotations are attributed by participant role (M, mother; HW, health worker; CL, community leader; PM, policymaker), with a participant number for focus group participants, and by LGA. Recordings conducted in Tiv, Idoma, and Nigerian Pidgin were first transcribed in the language used during data collection and subsequently translated into English for analysis. Translation was undertaken with attention to preserving the contextual meaning of participants’ accounts. Where applicable, quotations originally recorded in Nigerian Pidgin are presented in standard English translation to ensure accessibility for a wider readership. For participant feedback, 12 participants were invited to review relevant transcripts and comment on the preliminary interpretations: three mothers and three health workers, with one participant from each stakeholder group selected from each LGA, and all three policymakers and all three community leaders. Participants’ feedback was used to reflect on and refine the preliminary interpretations, and no substantive disagreements were raised. Reporting follows the Consolidated Criteria for Reporting Qualitative Research (COREQ) (26), with the completed checklist provided as Supplementary Table S1.

### Data analysis

Data were analysed using reflexive thematic analysis, following the six-phase framework developed by Braun and Clarke (27), which comprises familiarisation with the data, generating initial codes, searching for themes, reviewing themes, defining and naming themes, and producing the final report. The analysis was iterative and reflexive, with the researchers moving between the data, codes, and developing themes throughout the analytic process. More than one researcher independently engaged with the data, coding transcripts and generating initial interpretations, after which these interpretations and the developing themes were discussed within the research team and refined iteratively. This process was intended to support reflexive engagement with the data and transparency of interpretation, rather than to establish inter-coder reliability or measure coder agreement. Data were analysed using an inductive and iterative approach consistent with reflexive thematic analysis (27, 28). Data were managed and analysed in Microsoft Word. Given the modest size of the dataset (six focus group discussions and six individual interviews), specialist qualitative software was not considered necessary, and reflexive thematic analysis does not depend on software but on the researcher’s sustained interpretive engagement with the data (28). Word was also accessible to all members of the multilingual research team in Nigeria and the United Kingdom, enabling shared analysis across sites. Transcripts were organised by stakeholder group and study location; codes were recorded as comments on the transcripts and compiled into coding tables, which were used to develop and compare themes across stakeholder groups and LGAs. Coding decisions and changes in interpretation were documented in these tables and in analytic memos, forming the audit trail described below.

The analysis was conducted separately for each stakeholder group before undertaking cross-stakeholder comparison. Transcripts from mothers, health workers, policymakers, and community leaders were initially analysed independently within their respective stakeholder groups to identify patterns, meanings, codes, and developing themes specific to each group. Following the stakeholder-specific analyses, developing themes from the four groups were subsequently compared across stakeholder groups and across the three LGAs. This cross-stakeholder comparison examined areas of convergence, divergence, and stakeholder-specific emphasis while retaining important differences in participants’ experiences and perspectives.

Where researchers differed in their interpretation of codes or developing themes, the relevant transcripts were revisited, and the interpretations were discussed within the research team, with these discussions informing further refinement of the developing themes. Triangulation was undertaken during the interpretive stage by comparing findings across the two data-collection methods (focus group discussions and individual interviews), the four stakeholder groups, and the three LGAs. Convergent accounts were considered in refining shared themes, while divergent and stakeholder-specific accounts were retained to capture differences in experience, role, and context. An audit trail was maintained throughout the analysis to document coding decisions, changes in interpretation, theme development, refinement of themes, and key analytic discussions within the research team.

Reflexivity was considered throughout data collection and analysis. The research team comprised Nigerian and UK-based researchers who worked together across the three study locations. The researchers had no prior personal or professional relationships with participants. Data collection was undertaken by trained study team members who engaged with mothers, health workers, policymakers, and community leaders in the participating LGAs. The team considered how their professional positions, cultural and institutional backgrounds, and roles as researchers might influence interactions with participants, the accounts generated during the interviews and focus group discussions, and subsequent interpretation. These issues were discussed within the research team during the analytic process, particularly when developing and refining codes and themes, and relevant reflections were documented in analytic notes. This process helped the team remain attentive to how their positions and interpretations shaped the analysis rather than treating the themes as independent of the researchers’ engagement with the data.

### Ethical considerations

Ethical approval was obtained from the University of Manchester UREC committee Ref: 2026-25211-45551 and the Ministry of Health—Government of Benue State of Nigeria (MOH/STA/204/I/199/2026) also granted ethical approval for the study. This multi-level approval process ensured that the study met both international and local ethical standards. All methods were carried out in accordance with relevant guidelines and regulations, including the Declaration of Helsinki. All participants were competent adults capable of providing informed consent; therefore, consent from legal guardians or next of kin was not required. Informed consent was obtained directly from all participants before data collection. This comprised written informed consent for literate participants and witnessed oral informed consent for participants unable to read or write, with the witness signing on their behalf, in accordance with local and institutional ethics approvals. Given the potentially sensitive nature of discussions concerning flooding, household loss, psychological distress, and family disruption, a distress and safeguarding protocol was implemented during data collection to guide the research team in responding to any participant distress or disclosure of risk. The protocol included procedures for providing immediate emotional support, assessing distress and risk, involving the research team supervisor, and contacting appropriate health or safeguarding services where further support or action was required. Participants were also provided with information on available mental health support services and helplines where needed. The protocol was available throughout data collection; however, no participant experienced distress or disclosed a safeguarding concern that required referral or additional support. This approach ensured that participation remained voluntary and that appropriate support was available if required.

## Results

This section presents findings from focus group discussions with mothers and health workers and one-to-one interviews with community leaders and policymakers across three local government areas of Benue State. Across participant groups, flooding was described as a recurring climate-related stressor that disrupted household routines, caregiving, livelihoods, service access, and child wellbeing, while mothers and health workers also described extreme heat as an additional source of strain. Analysis generated five themes: (1) flooding exposure and spatial–infrastructural vulnerability; (2) household disruption and caregiving strain; (3) reported maternal psychological distress under climate stress; (4) early childhood development implications; and (5) coping, protection, and response gaps. Each theme is presented below and illustrated with participant quotations.

### Flooding exposure and spatial-infrastructural vulnerability

Participants generally framed flooding as a regular aspect of life in their respective communities and described limitations in anticipation and management. For example, mothers and community leaders described flood onset as seasonal but disruptive, while policymakers mentioned that certain local government areas are often impacted due to their unique geographic and infrastructural peculiarities. As one participant said: *“The flood situation in my area… the frequency is high, because every year when it rains there is bound to be flood”* (CL, Otukpo). Likewise, a policymaker noted, *“We have a trend… some local governments [i.e., Makurdi] are usually affected… when the flooding comes, it affects them mostly”* (PM, Makurdi). The spatial concentration of risks was often associated with settlement location, land access, and housing quality. Flood-prone communities were described as low-lying, river-bound, and usually occupied by low-income families. As one community leader elucidated, *“Persons who could not afford to acquire lands in better places… purchase it in those areas”* (CL, Gboko). This position was reinforced by another participant, indicating that *“…some houses are built blocking channels where water flow… when rain comes heavily there is no passage”* (PM, Gboko).

Many participants associated the severity of flooding with infrastructural conditions, including inadequate drainage, poor road networks, and congested or obstructed channels. As one community leader remarked: *“When there is rain, there is no street [drainage]… no way water will go… water will just stand still”* (CL, Makurdi). Another participant added, *“There is no drainage, water must flow… but it becomes a problem when the flood comes in”* (CL, Otukpo).

These accounts linked flood vulnerability to environmental management conditions, including inadequate drainage, poor roads, and obstructed channels, alongside heavy rainfall. Other participants equally linked infrastructure failures to service access, indicating that inaccessible roads prevented both patient movement and provider mobility. As one participant noted, *“There will be no access… there is no way they can come into the hospital”* (HW3, Otukpo).

Across the data, participants described flooding as something made worse by where people live, poor planning, and weak environmental infrastructure, and as a recurring reality of daily life that they linked to wider household and health consequences.

### Household disruption and caregiving strain

Participants described experiencing household-level flooding as a compound shock that disrupted food, shelter, mobility, work and access to services. During discussions, several mothers described losing beds, clothes, and domestic space, while the community leaders reported that families sometimes slept outside. As one participant mentioned, *“Our bed, wardrobe, everything has spoiled because of the flood”* (M3, Makurdi). Another participant also observed: *“You will see people outside sleeping… with their mattress and children”* (CL, Makurdi). These accounts illustrate how flooding extended beyond property damage to affect where families could live and sleep, including through temporary exposure outside the home.

Several participants also spoke of the economic consequences of flooding. They described crop loss, disrupted trading, and the inability to work during periods of flooding. As one policymaker explained, *“You have put all your income in the farm, and the flood wipes it away… you are back to square one”* (PM, Makurdi). For participants engaged in informal trade, flooding was associated with reduced daily earnings and difficulty securing food for the household. One participant noted, *“We cannot go out to do our normal business… it makes us hungry”* (M3, Otukpo). These accounts suggest that the consequences of flooding extended beyond physical damage to reduced earnings and difficulty restoring household resources.

Participants described food and water insecurity as closely linked to these economic losses. Many participants described how water sources, food stocks, and cooking were, respectively, contaminated, destroyed and compromised in flooded homes. One participant stated: *“We cannot find clean water to drink… there is no clean water to use for cooking”* (M8, Otukpo), while a community leader remarked: *“You have to manage with whatever water you can find”*. (CL, Makurdi). Participants described these conditions as creating concerns about health and increasing the burden on caregivers who had to secure safe food and water under constrained circumstances.

Participants described caregiving during flooding and extreme heat as more labour-intensive and difficult to maintain. They narrated the effort put into locating food, protecting children, and sustaining domestic routines under uncertain situations. As one participant elucidated, *“You have to struggle to get food before your children can eat”* (M7, Makurdi). Another said that food preparation could turn impossible when a home is submerged in flood: *“I could not cook because all the food I had prepared was submerged in water”* (M8, Makurdi). There were disruptions to nighttime routines, as one participant noted, *“During the rainy season, I do not sleep at night. Whenever it starts raining, I cannot sleep again”* (M5, Makurdi). Extreme heat contributed additional strain, particularly for mothers looking after children: *“My children will not sleep from night till daybreak. I will be fanning them”* (M3, Makurdi).

Overall, participants described substantial disruption to household routines, with caregiving becoming more demanding and household resources more difficult to secure.

### Reported maternal psychological distress under climate stress

Participants described experiences of psychological distress associated with flooding, extreme heat, repeated household disruption, economic loss, uncertainty, and the challenges of caring for children under unstable conditions. These accounts represent participants’ descriptions of emotional and psychological strain rather than clinically assessed maternal mental health outcomes. Several mothers described anticipatory worry, particularly when rainfall signalled the possibility of renewed flooding. One mother said, *“When the clouds gather… you say, ‘Oh! It is going to rain again,’ and you start worrying about your house”* (M6, Makurdi). This account indicates that anticipation of another flood was accompanied by worry extending beyond the immediate flood event. Participants also described heat-related discomfort, poor sleep, and exhaustion alongside concerns associated with flooding. As noted above, one mother described staying awake at night to fan her children (M3, Makurdi), and a health provider explained that *“Excess heat causes mothers’ sleepless nights because the babies they are caring for are uncomfortable”* (HW2, Gboko).

Fear and emotional strain were highlighted by community leaders, who associated anxiety and instability among mothers with flooding. As one participant said: *“This is the main stress of flood…, it instils fear in mothers’ mind… they no longer stay stable”* (CL, Gboko). Across several FGDs, participants linked maternal distress to concerns about children’s wellbeing as well as household conditions. As one participant put it, *“The children cry to you, saying, ‘Mummy, I want to eat,’ and you have nowhere to go to get food”* (M1, Makurdi). Health providers also highlighted this relational level of stress, observing that *“Mother is always thinking how she will do and the children will be okay”* (HW4, Makurdi). Overall, participants described maternal psychological distress as arising from the combined pressures of flooding, heat, hunger, economic disruption, and caregiving demands. Participants also described economic losses as an additional source of psychological strain. Participants described the loss of farms, livelihoods, and household assets as producing repeated setbacks and a sense of uncertainty. One participant captured this succinctly, *“Household items are damaged, food items are also washed away from within the houses and from the farmlands that we have around the areas… You see, the farmland is all washed… The income, or let me say the means of livelihood of the parents, is affected because they could not go to the market anymore. And even those that sell within the houses, they cannot sell anymore”* (PM, Otukpo). Participants also described these pressures as affecting family stability and relationships. For example, one community leader observed: *“This flooding has caused some of our wives to leave us because women do not like to suffer”* (CL, Makurdi). These accounts suggest that participants perceived climate-related hardship as extending beyond personal distress to affect domestic and relational life.

Participants described limited recognition of and support for maternal psychological difficulties, with stigma and cultural beliefs identified as potential barriers to seeking formal mental health care. One participant explained: *“Not all mothers talk about it… some keep it within them… they do not disclose it”* (HW4, Makurdi). Participants further pointed to stigma and cultural barriers to seeking mental health help: *“Some people attach it to traditional belief… instead of going for medical treatment”* (PM, Makurdi).

### Early childhood development implications

Participants described climate-related disruption as affecting not only children’s immediate health but also the broader conditions necessary for early childhood development. Participants described several ways in which flooding and related household disruptions could affect conditions that support early childhood development. These accounts centred on nutrition, illness exposure, access to healthcare and immunisation, continuity of learning, safety, caregiving, and opportunities for stable social interaction. These domains were closely interconnected, with disruption in one area often affecting others.

Nutrition emerged as a prominent concern in participants’ accounts. Mothers reported challenges feeding children appropriately after flooding has damaged food supplies or limited access to safe water. As one participant stated: *“…it even causes hunger; it makes us hungry because we cannot find food to eat, and we do not have money to buy those things”* (M5, Otukpo). Health workers and community leaders associated these conditions with malnutrition among children. One health worker observed, *“Most children cannot feed well… we will experience children malnutrition because of the feeding pattern and health challenges”* (HW3, Makurdi). A community leader (CL, Makurdi) also noted that inadequate food and unsafe water contributed to conditions such as kwashiorkor, a severe form of protein-energy malnutrition characterised by oedema and impaired growth. These accounts suggest that flooding can disrupt food and water conditions that participants viewed as important for children’s wellbeing and development.

Participants equally highlighted increased exposure to illness. As one stated: *“When you are not feeding properly… malnutrition may come in, and the effect of it is seen in the hospital”* (PM, Makurdi). This account illustrates how participants linked inadequate nutrition with concerns about children’s health. Another repeated issue was access to healthcare. As one participant explained, *“Patients… cannot make it to the clinic because of bad roads”* (HW8, Gboko). Participants also linked flooding to disruption of preventive services: *“Services will be denied… even routine immunisation”* (PM, Otukpo). Participants described how physical access barriers could delay treatment and disrupt access to routine child health services.

Participants described disruptions to learning and social routines during periods of flooding, including prolonged school disruption. As one elucidated: *“Anything from August, you cannot see children going to school… no way”* (CL, Makurdi). Beyond schooling, participants described displacement as disrupting children’s familiar routines, caregiving arrangements, and social support networks. Participants viewed these disruptions as important because child development depends not only on physical wellbeing but also on continuity in learning, caregiving, and social engagement.

Participants also raised safety concerns for young children. Community leaders described floodwaters as creating dangerous living environments, particularly for young children. One participant stated, *“The floodwater could carry away our small children”* (CL, Makurdi). Others highlighted the insecurity associated with displacement and temporary shelter arrangements. Overall, participants portrayed flooding as creating a developmental environment characterised by multiple and overlapping risks, affecting children’s health, learning, safety, and social stability simultaneously.

### Coping, protection, and response gaps

Despite the challenges linked to flooding and other climate-related stressors, participants described coping mechanisms and support strategies that helped households manage these disruptions. At the community level, participants reported local collective action. Particularly, community leaders described mobilising residents to clear blocked drainage channels and enhance water passage. As one explained: *“I used to mobilise youth to clear road… but I cannot dig well for everybody”* (CL, Makurdi). Another narrated the community effort to *“create pathway for floodwater drainage”* (CL, Gboko). In addition to illustrating local resilience, these accounts highlight the limitations of community-led action in the absence of more extensive infrastructure support.

Participants described social and religious networks as important sources of assistance. Several participants described how neighbours, family members, and community members offer temporary shelter, food, and useful assistance during flooding events. As one participant stated, *“Neighbours can go and help… those that are on dry land, they can help out”* (CL, Otukpo).

Policymakers were the main individuals who talked about formal response mechanisms. Participants discussed preparatory initiatives, relief distribution, and emergency coordination systems. *“We have WASH in the Emergency Technical Working Group… when there is flooding we respond in terms of awareness, movement of people to safe places, and preparedness,”* a policymaker noted (PM, Makurdi). Another detailed how *“flood-relief supplies, food, and clothing were given to impacted households”* (PM, Makurdi). These reports indicate that formal response mechanisms were in place, while participants also described variation in their reach and implementation.

Health providers and policymakers underscored operational challenges across formal systems. One of them noted that *“Most of these activities… the Ministry of Health is not carried along… there is lack of collaboration”* (PM, Makurdi). They linked this lack of collaboration to difficulties in coordinating services during flood events.

Participants also described service delivery gaps, particularly in relation to healthcare access during flooding. Health workers and policymakers described shortages of personnel, logistical challenges, and limited capacity to reach affected populations: *“Health personnel are not sufficient… when there is flood you need more staff… logistics… is a challenge”* (PM, Makurdi). Participants also described geographic barriers, particularly in rural and hard-to-reach areas, where access sometimes required improvised solutions such as boats.

Importantly, participants also highlighted the absence of long-term and sustainable interventions, particularly in relation to resettlement and recovery. Community leaders emphasised that while short-term relief may occasionally be provided, there is little evidence of sustained support: *“What about the medium term or even the long term… before they are finally resettled… these are virtually not in existence”* (CL, Otukpo). Participants described recurring flooding as requiring repeated short-term coping in the absence of sustained structural support.

## Discussion

This study explored how participants experienced flooding and extreme heat in relation to household vulnerability, maternal psychological wellbeing, caregiving, and perceived implications for early childhood development in Benue State, Nigeria. Across the stakeholder groups, flooding was described as an interconnected stressor affecting household routines, livelihoods, food and water security, service access, and caregiving, while mothers and health workers also described extreme heat as disrupting sleep and caregiving. Participants also described emotional distress among mothers and multiple disruptions affecting young children. These findings should be interpreted as accounts of lived experience and perceived effects rather than as direct measurements of maternal mental health or child developmental outcomes.

Participants associated flooding with inadequate planning, infrastructure deficits, and the location of households in flood-prone areas, rather than describing it solely in terms of rainfall. This aligns with Nigerian and African evidence that flood exposure is concentrated by infrastructural deficits and spatial inequality (1, 19), and points to the relevance of anticipatory risk reduction alongside post-event relief, consistent with approaches described in other Global South settings through drainage rehabilitation, participatory planning, and climate-resilient land-use governance (29–32).

At the household level, participants described how flooding disrupted domestic life through property damage, water contamination, food loss, livelihood interruption, displacement, and sleep disruption. Their accounts further described caregiving as becoming more physically and mentally demanding as mothers simultaneously managed food, hygiene, and children’s distress. Similar gendered burdens have been reported in other flood-affected settings, where mothers carried primary responsibility for protecting children, securing food and water, and cleaning homes during and after floods (33, 34). Studies from comparable settings also report limited household-level planning for water and food ahead of floods, with coping strategies that are largely reactive and short-term (35). Together, these findings suggest that household-level preparedness across water, food, shelter, and transport warrants greater attention within flood response.

Stigma and cultural interpretations of distress were described as barriers to seeking formal mental health support, contributing to the limited recognition of maternal psychological needs within formal systems. Participants also described how flooding disrupted several conditions relevant to children’s wellbeing and early development, including food security, access to safe water and healthcare, school attendance, opportunities for social interaction, and stable caregiving. These accounts suggest that climate-related disruptions can affect multiple aspects of children’s everyday lives simultaneously. Consistent with evidence linking maternal distress and disruptions in maternal–child interaction with adverse developmental and nutritional experiences (12, 13), the findings highlight the interconnected nature of maternal wellbeing and the environments in which young children live, learn, and receive care. Given the importance of continuity, safety, and responsive caregiving during early childhood, flood-response measures could usefully extend beyond immediate illness management to support nutrition, routine healthcare, early learning, safe environments, and nurturing care.

Despite these pressures, participants described communities as actively responding: residents cleared drainage, shared resources, and mobilised social and religious networks to help rebuild. Participants described many community responses as short-term and reported limitations in the reach, continuity, and coordination of formal responses. Evidence and programme experience from comparable settings support the use of anticipatory action, early warning systems, and community-based preparedness as components of disaster preparedness (36, 37), while task-sharing approaches for caregiver mental health show promise through community health worker home-visiting models in low-resource settings (38). These findings support consideration of integrated approaches that combine early warning, maternal mental health support, nutrition, and child protection rather than addressing these areas solely through separate programme streams.

The composition of the health-worker groups also varied across the three LGAs in terms of professional cadre, qualifications, facility type, and demographic characteristics. These differences may have influenced the emphasis placed on service access, clinical capacity, and institutional response within participants’ accounts. Accordingly, differences in health-worker narratives across LGAs should be interpreted in light of both local conditions and the composition and professional roles of participants represented at each site.

The findings also support consideration of climate adaptation as an important health-system concern in Benue, consistent with WHO guidance on climate-resilient health systems and health-focused adaptation planning (39). Maternal and child health services could embed flood-continuity planning through rapid response mechanisms and community-based systems (34, 37). Psychosocial support also could be integrated into routine antenatal, postnatal, and child health services through culturally adapted, task-shared, frontline-delivered models (40). Targeted social protection, including cash transfers, may complement emergency responses to climate-related hazards by reducing household vulnerability and supporting recovery (41, 42). Across all of these, co-design with mothers, community leaders, health workers, and local officials could help align interventions with lived experience and local priorities.

## Strengths and limitations

The study was conducted in a single state, and although Benue’s high flood exposure makes it an important setting, experiences may differ in other regions of Nigeria and in other climate-affected contexts. All community leaders and policymakers who participated were male, and fathers’ perspectives on caregiving and household stress were not captured, meaning the findings offer a partial view of gendered roles within flood-affected households. The findings rely on self-reported accounts, which may be shaped by recall and social desirability, particularly in descriptions of institutional responses. Data were collected and translated across four languages, and although steps were taken to preserve meaning, some nuance may have been lost; member checking was also limited to a subset of participants. In addition, the exploration of extreme heat was not uniform across stakeholder groups. Mothers and health workers were explicitly asked about extreme heat, whereas the topic guides for policymakers and community leaders focused primarily on flooding, infrastructure, and institutional response. Consequently, findings related to extreme heat are informed predominantly by mothers’ and health workers’ perspectives and should not be interpreted as representing equivalent cross-stakeholder exploration of heat-related experiences. Finally, maternal mental health was explored through participants’ own descriptions rather than validated measures, which limits comparability with clinical and epidemiological studies but reflects the study’s aim of understanding lived experience. Child developmental outcomes were not directly assessed using validated developmental instruments or other objective measures. Accordingly, the findings reflect participants’ reported experiences and perceived implications for children’s wellbeing and development, rather than measured developmental effects. The study also did not include objective measurements of flood or heat exposure or a formal climate-attribution analysis. It therefore cannot establish whether the flooding and heat events described by participants were directly attributable to climate change; rather, it documents participants’ experiences of these events and their perceived consequences.

## Conclusion

Climate-related flooding and extreme heat were described as important sources of disruption for mothers, young children, and households in Benue State. Participants described how flooding was intertwined with household disruption, livelihood losses, caregiver stress, maternal psychological distress, and challenges affecting children’s nutrition, health, learning, and safety. Consistent with evidence that climate change disproportionately affects marginalised populations (1, 19), Participants’ accounts portrayed flooding not simply as a discrete event but as a recurring stressor experienced alongside pre-existing inequalities in land, infrastructure, and economic security.

A key contribution of this research is foregrounding maternal psychological distress as an important component of participants’ accounts of climate-related disruption. Participants also described multiple and interconnected challenges affecting the conditions in which young children live, learn, and receive care. These findings highlight the importance of moving beyond narrowly focused emergency responses towards approaches that strengthen infrastructure and land-use governance (29, 32), maintain maternal and child health services during flood periods (34, 37), integrate appropriate psychosocial support within primary care (40), and strengthen social protection during periods of disruption (41, 42). Such approaches could be informed through co-design with affected communities and grounded in climate-resilient health-system planning (39).

## Data Availability

The datasets presented in this article are not readily available because participant confidentiality and ethical restrictions protect sensitive narrative accounts. Requests to access the datasets should be directed to jahanara.miah@manchester.ac.uk.
